# Supplementary figures and images for: Emergence of artemisinin-resistant Plasmodium falciparum with kelch13 C580Y mutations on the island of New Guinea
Source: PLoS Pathog. 2020 Dec 15;16(12):e1009133. doi: 10.1371/journal.ppat.1009133 (PMC7771869; doi:10.1371/journal.ppat.1009133)

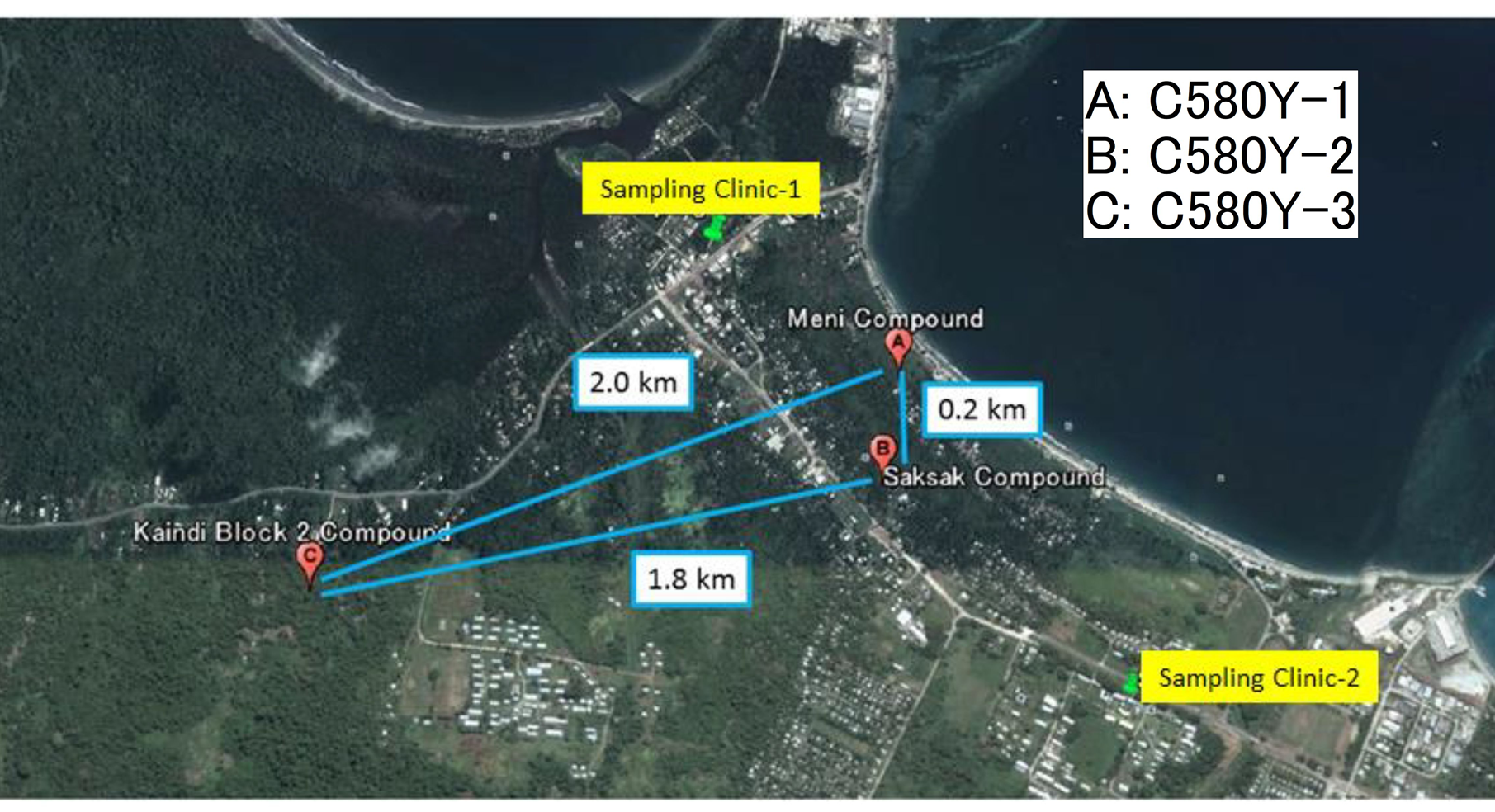

Supplement: S1 Fig — This map of Wewak town shows the place of abode of the three patients whose parasites carried the kelch13 C580Y allele (red markers), the distance between these locations (blue lines), and the location of the two clinics where the study was carried out (green markers) (TIF) [file ppat.1009133.s001.tif]

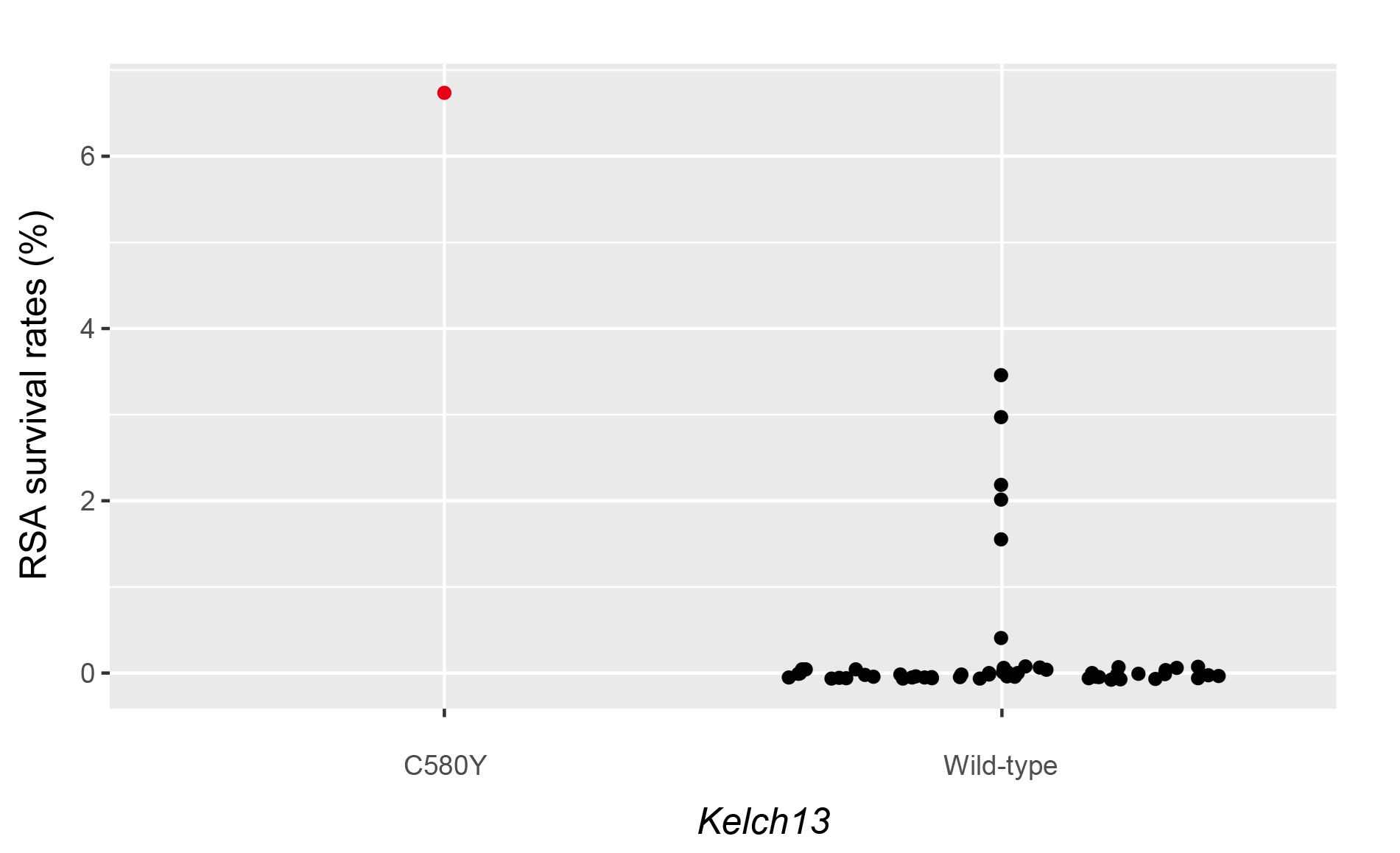

Supplement: S2 Fig — The plot compares the RSA survival rate (see Methods) of one of the Wewak kelch13 C580Y mutants (left, red marker) against those for wild-type parasites from the same area (right, black markers). RSA survival rates could not be determined for the remaining two Wewak kelch13 C580Y mutants. Artemisinin-susceptible laboratory clone 3D7 showed no parasite at 700 nmol/L. MRA-1236 and MRA-1240 (artemisinin-resistant laboratory clones) showed survival rates 14.3% and 27.0%, respectively. (TIF) [file ppat.1009133.s002.tif]

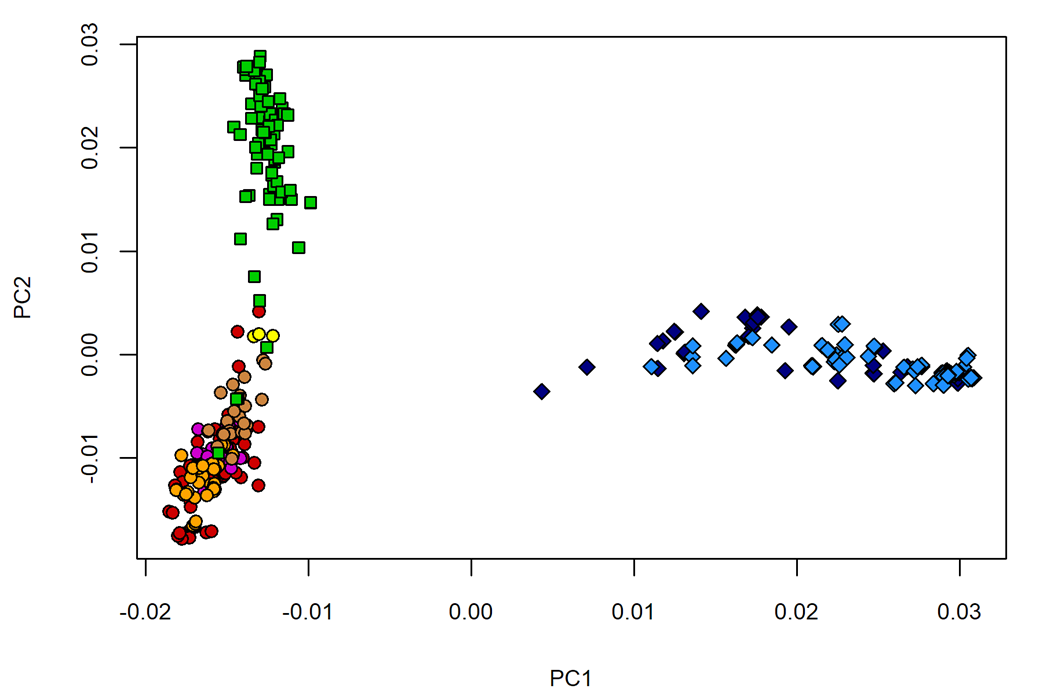

Supplement: S3 Fig — This figure shows a plot of the first two components, for all analyzed samples. The first component (PC1), which explains most of the dataset variance, separates samples from Cambodia from those in New Guinea. The second component (PC2) separates parasites from Papua Indonesia from those found in PNG. We note that there is some overlap between these two groups, and the Wewak C580Y parasites (yellow) appear at an intermediate point between PNG and Indonesian parasites. (TIF) [file ppat.1009133.s003.tif]

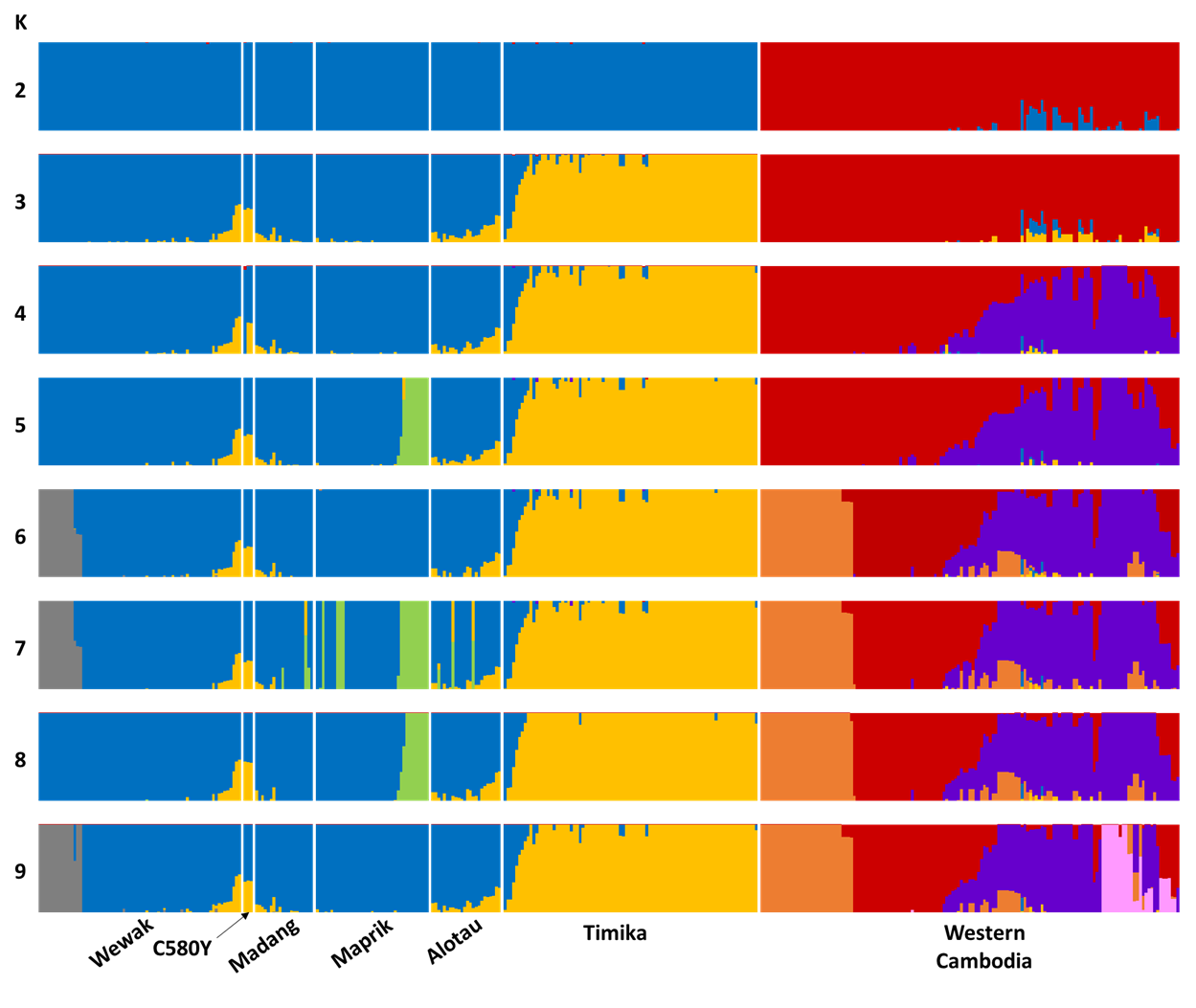

Supplement: S4 Fig — The plots show admixture levels estimated by the fastSTRUCTURE software, based on the hypothesis of K ancestral populations (K = 2 to 9, shown on the left-hand side of the plot). Each population is represented by a different colour (K arbitrary colours for each plot); each sample is shown as a vertical bar, coloured according to the proportion of ancestry from each population. Samples are grouped by sampling location, as shown by labels at the bottom; Wewak C580Y mutants are shown as a separate group. From an analysis of the underlying data, the fastSTRUCTURE chooseK tool reported that K = 6 is that number of populations that best explains the population structure. (TIF) [file ppat.1009133.s004.tif]

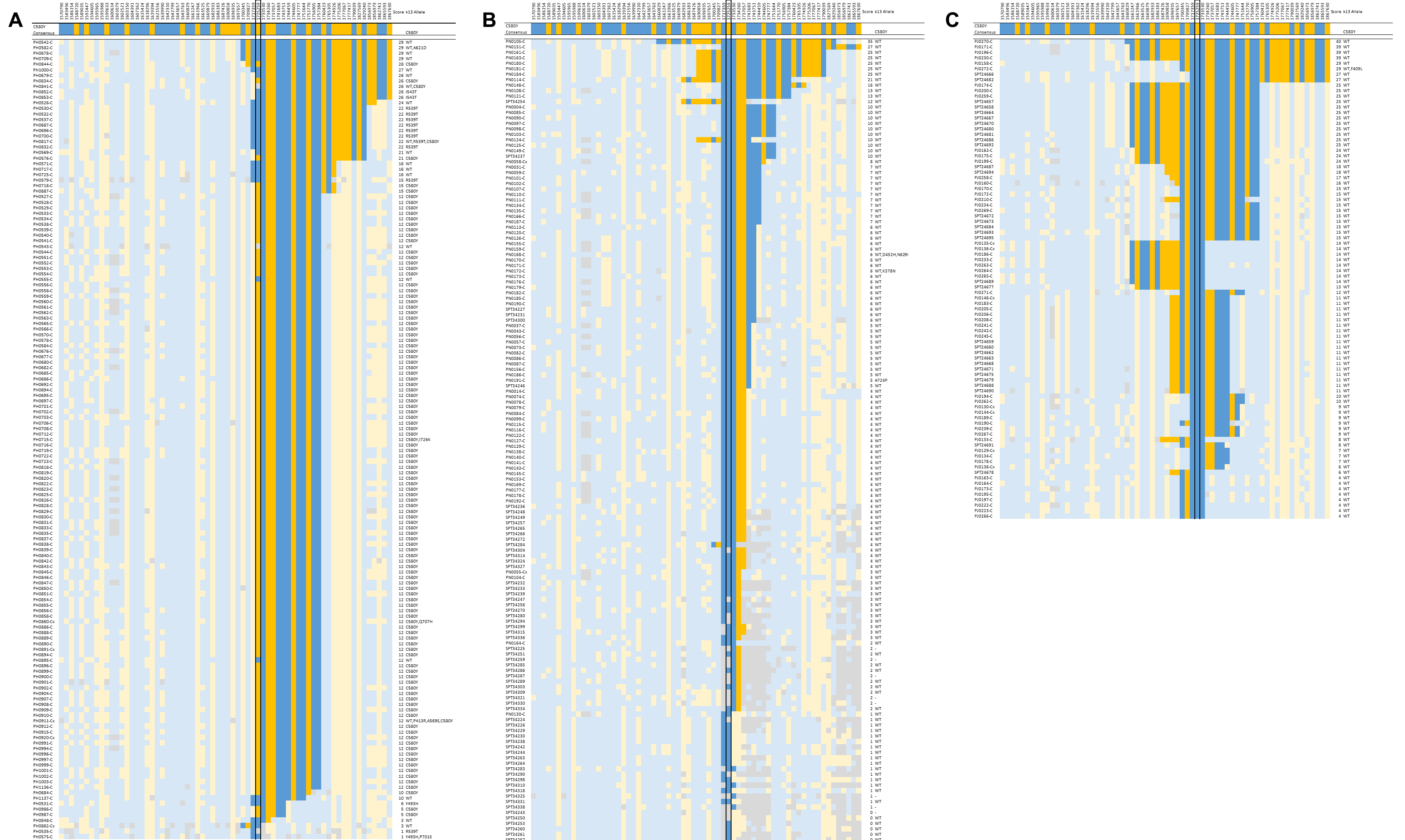

Supplement: S5 Fig — Each row represents the haplotype of a sample, the top row showing the consensus haplotype for the C580Y samples in Wewak. Each column represents a variant position; the position of the kelch13 C580Y mutations (1725259) is indicated by a box outline. Cells colours show the allele call at each position in the sample. Deep colour hues indicate a matching haplotype portion (i.e. consecutive positions within the flanking haplotypes that match the consensus haplotype), while lighter colours indicate positions after a haplotype mismatch. Blue cells symbolize the reference allele, orange the alternative allele, and gray denote a mixed allele call or insufficient coverage. Samples are grouped by country of provenance: Cambodia (panel A), Papua New Guinea (B), Indonesia (C), and sorted by decreasing matching score (the sum of length of the matching haplotype portions in the two flanks). The column on the left shows the sample identifier, while the columns on the right show the matching score and the kelch13 allele carried by the sample. (TIF) [file ppat.1009133.s005.tif]

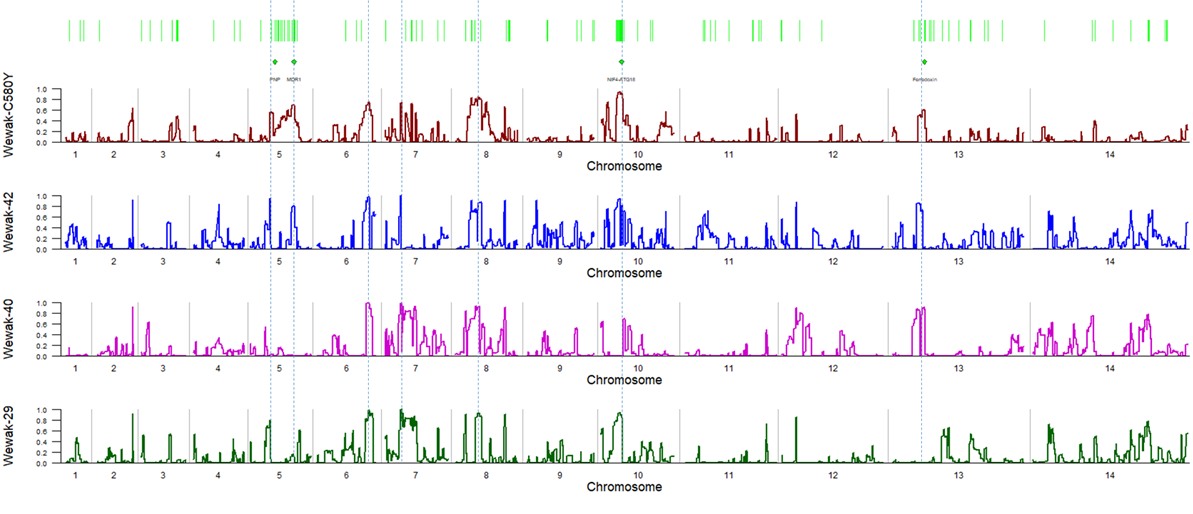

Supplement: S6 Fig — These plots show, across all nuclear chromosomes, the proportion of IBD pairs between Timika and the Wewak C580Y mutants (red, top), and three Wewak samples that showed a high proportion of common ancestry with Timika: one with 42% Timika ancestry (blue), one with 40% (magenta) and one with 29% (green). The top panel shows vertical green bars marking highly differentiated positions where the C580Y mutant carry a Timika-like allele. Green diamond markers show the location of some notable drug resistance-related alleles identified in our analysis. Vertical dotted lines act as visual guides to show correspondences. (TIF) [file ppat.1009133.s006.tif]
