# Supplementary material for: Emergence of artemisinin-resistant Plasmodium falciparum with kelch13 C580Y mutations on the island of New Guinea
Source: PLoS Pathog. 2020 Dec 15;16(12):e1009133. doi: 10.1371/journal.ppat.1009133 (PMC7771869; doi:10.1371/journal.ppat.1009133)
Supplement: S1 Text — A description of the climatic, entomological and epidemiological characteristics of the Wewak location, with a description of recent malaria control interventions. (DOCX) [file ppat.1009133.s015.docx]

# Supplementary Notes

## Description of sampling locations

Wewak Town comprises about 25,000 inhabitants. The average temperature in the studied area is 27.3°C (min 23.8°C, max 30.9°C) with an annual rainfall of approximately 3000 mm. All four species of human malaria parasites were observed with limited seasonal variations between the wet (October to April) and dry (May to September) seasons, and were transmitted mainly by *Anopheles farauti,* *Anopheles punctulatus*, and *Anopheles koliensis* [1-3].

Free distribution of long-lasting insecticidal mosquito nets (LLIN) was implemented by The Government of PNG between 2005 and 2009 and between 2009 and 2013 [4]. Average LLIN usage was 55% in 2008 and 2009 and 32.9–67.7% in 2013–2014 [5, 6]. *P. falciparum* entomological inoculation rates in Dreikikir, about 50 km from our study area, dropped from 159 infective bites/person/year in 2008 to 53 in 2011, following LLIN distribution.[7] Current first-line regime is Artemether plus Lumefantrine, officially introduced in 2010. Free distribution of ACT and rapid diagnostic tests (RDT) through all public health facilities was initiated by the malaria control programme in late 2011 [4]. Previously, intramuscularly artemether was sometimes used in patients who failed first-line treatment regime.

# References

1. Muller I, Bockarie M, Alpers M, Smith T. The epidemiology of malaria in Papua New Guinea. Trends Parasitol. 2003;19(6):253-9. Epub 2003/06/12. PubMed PMID: 12798082.

2. Schultz L, Wapling J, Mueller I, Ntsuke PO, Senn N, Nale J, et al. Multilocus haplotypes reveal variable levels of diversity and population structure of Plasmodium falciparum in Papua New Guinea, a region of intense perennial transmission. Malar J. 2010;9:336. Epub 2010/11/26. doi: 10.1186/1475-2875-9-336. PubMed PMID: 21092231; PubMed Central PMCID: PMC3002378.

3. Barry AE, Schultz L, Senn N, Nale J, Kiniboro B, Siba PM, et al. High levels of genetic diversity of Plasmodium falciparum populations in Papua New Guinea despite variable infection prevalence. Am J Trop Med Hyg. 2013;88(4):718-25. doi: 10.4269/ajtmh.12-0056. PubMed PMID: 23400571; PubMed Central PMCID: PMC3617858.

4. Hetzel MW, Pulford J, Maraga S, Barnadas C, Reimer LJ, Tavul L, et al. Evaluation of the Global Fund-supported National Malaria Control Program in Papua New Guinea, 2009-2014. P N G Med J. 2014;57(1-4):7-29. Epub 2014/03/01. PubMed PMID: 26930885.

5. Hetzel MW, Pulford J, Ura Y, Jamea-Maiasa S, Tandrapah A, Tarongka N, et al. Insecticide-treated nets and malaria prevalence, Papua New Guinea, 2008-2014. Bull World Health Organ. 2017;95(10):695-705B. Epub 2017/11/18. doi: 10.2471/BLT.16.189902. PubMed PMID: 29147042; PubMed Central PMCID: PMC5689189.

6. Hetzel MW, Gideon G, Lote N, Makita L, Siba PM, Mueller I. Ownership and usage of mosquito nets after four years of large-scale free distribution in Papua New Guinea. Malaria journal. 2012;11(1):192.

7. Reimer LJ, Thomsen EK, Koimbu G, Keven JB, Mueller I, Siba PM, et al. Malaria transmission dynamics surrounding the first nationwide long-lasting insecticidal net distribution in Papua New Guinea. Malar J. 2016;15(1):25. Epub 2016/01/13. doi: 10.1186/s12936-015-1067-7. PubMed PMID: 26753618; PubMed Central PMCID: PMC4709896.
