# Supplementary material for: Emergence of artemisinin-resistant Plasmodium falciparum with kelch13 C580Y mutations on the island of New Guinea
Source: PLoS Pathog. 2020 Dec 15;16(12):e1009133. doi: 10.1371/journal.ppat.1009133 (PMC7771869; doi:10.1371/journal.ppat.1009133)
Supplement: Attachment — Submitted filename: ResponseToReferees.pdf [file ppat.1009133.s016.pdf]

Miotto O, et al. Emergence of artemisinin-resistance Plasmodium falciparum with kelch13 C580Y mutations on the island of New Guinea

Responses to Referees' Comments

Dear PLOS Pathogens Editors,

We would like to thank the reviewers for their thorough analysis of the manuscript, and for the many thoughtful comments they provided. We have addressed these comments to the best of our abilities, and we attach a revised manuscript (including a version with change bars) for your consideration.

This document contains point-by-point responses to the reviewers' comments, which explain the changes to the manuscript. We trust they will satisfy the editors as well as the referees.

Thank you again for your interest and support.
Yours sincerely,
Olivo Miotto (Corresponding author)

Reviewer 1

Part III – Minor Issues: Editorial and Data Presentation Modifications

Some part of the narrative seems a bit like story telling. The high Fst SNPS that were introgressed being associated with resistance. What is the Fst distribution between Indonesia and PNG?
What is the extent of the evidence that the high Fst SNPs are likely to have been selected in Indonesia, could they be introgressed from elsewhere? Are there other possible sources of introgression. I think the ambiguity about evolutionary scenario is somewhat inevitable since really it hinges on the demographic history in Indonesia and elsewhere which is not the topic of the present manuscript. It should be made as clear as possible which part of this argument is well supported by data and which is more indirect inference. Most important, what is the evidence that this association with resistance, which is interesting if true whatever the demographic explanation is really statistically surprising?

> The question of whether there is evidence indicating evolutionary selection of the introgressed segments in Papua Indonesia was asked by both reviewers and seems highly pertinent.
>
> Papua Indonesia has been known to harbour high levels of multidrug resistant *P. falciparum* and *P. vivax* (e.g. see Ratcliff A, *et al. Trans R Soc Trop Med Hyg* 2007; **101**: 351-9; Sumawinata IW*, et al*. *Am J Trop Med Hyg* 2003. **68**:416-20., and discussion in another response further below), and frontline treatment had to be changed to dihydroartemisinin-piperaquine. Even so, at population level there are signs of continued selective pressures: a recent study has shown that a distinct parasite subpopulation emerged after the change in treatment, and has expanded more or less clonally, accounting for 100% of infections in 2016-2017 (Pava Z et al., *PLoS Negl Trop Dis* 2020; **14**(5): e0008295).

The Timika genomic data analysed in our manuscript are part of a larger Indonesian study using genetic and clinical data; the identification of signatures of selections in the Timika genomes is therefore part of a separate study. Although the isolates evaluated to date do not have the *kelch13* C580Y mutations, or amplifications of *plasmepsin2*-3 copy number, it is possible that the Timika population is undergoing other selective changes that support the emergence of artemisinin and piperaquine resistance. The ongoing analyses of Indonesian parasites will bring further clarity to this question, but this is beyond the scope of the current analysis.

Although the definitive results are not yet at our disposal, we note that the introgressed segments have two important characteristics: (a) they are long (typically >100kbp, much longer than typical linkage disequilibrium decay- see Manske M, Miotto O, et al. *Nature* 2012; **487**(7407): 375-9); and (b) their haplotypes are shared by a high number of Timika parasites, which is reflected by the high IBD levels. Since high-frequency long haplotypes are the most commonly used signatures of recent selection (Sabeti PC, *et al*. *Nature* 2002; **419**(6909): 832-7), the evidence available is heavily suggestive that these regions are under selection. In our article, we had previously stated: “The sizes of these haplotypes (generally > 100 kbp) suggests they were recently acquired from a donor population- putatively, one circulating in Indonesian Papua- and possibly under evolutionary selection”. We have now clarified as follows:

> The sizes of these haplotypes (generally > 100 kbp) suggests they were recently acquired from a donor population- putatively, one closely related to those in Indonesian Papua. The high level of IDB in these regions indicates that a large proportion of Timika parasites possess the same long haplotype, which is characteristic of genomic regions under evolutionary selection.

A further point raised is that of the distribution of F_{ST} values. It appears that the reviewer assumes that we intended high F_{ST} values to be evidence of selection, but this is not the case. Starting from the assumption that these introgressed segments may have been acquired because they contain some beneficial variant, we sought to filter the variants contained in these sizeable regions to a smaller set of candidate SNPs that could be studied further. To identify plausible candidates, we leveraged the fact that the acquired haplotype was at high frequency in Timika but absent for Wewak. If the introgression was driven by the acquisition of a new variant, it seems reasonable that this variant should be at high frequency in Timika and at low frequency in Wewak, i.e. highly differentiated between the two populations. Applying a threshold of F_{ST}>0.3 produces a list of only 0.6% of all non-synonymous variants analysed in this study (n=144 of 24,216), providing a manageable list of potential candidates- the mutations that lie in the acquired regions.

It is important to point out, however, that a high degree of differentiation does not constitute evidence of selection, nor does it prove a particular allele has driven the introgression, or that it confers phenotypic changes associated with the *kelch13* mutations. Nevertheless, given that the parasites that acquired these segments also carry the C580Y ART-R mutation, it is reasonable to pinpoint, among the potential candidates, those that have previously been implicated in drug resistance by other studies and in other geographies. Although these variants are only “guilty by association”, we believe it is important to highlight them as prime candidates for further investigation. We have modified some sentences in the main text (under “Genetic variants association with *kelch13* C580Y”) to clarify these points as follows:

> Because of the length of the shared IBD segments, it is not possible to pinpoint exactly what variants may have driven their acquisition. However, it is possible to narrow down the list of potential candidates, based

> on the expectation that the driving mutations would cause amino acid changes, and would be common in Indonesian Papua and rare in PNG. Therefore, we extracted those nonsynonymous variants that are highly differentiated SNPs between the Wewak and Timika populations ($F_{ST} \geq 0.3$), and at which the C580Y mutants carry the Timika-like allele (n=144, 0.6% of all nonsynonymous variants), ordering them by decreasing F_{ST} (Supplementary Table 6). The resulting list of variants may be used as a shortlist for further functional investigations; it may be of particular interest that several of these variants, particularly those in regions of high IBD with Timika parasites, have been previously implicated in drug response, or are located in genes associated with drug resistance.

And in the Discussion:

> Although the available data does not allow precise identification of the genetic variants that drove the segments' acquisition, we catalogued alleles located in the IBD fragments that were shared with the Indonesian Papua parasites and absent from other PNG samples. In this shortlist of potential drivers, we found several variants previously associated with drug resistance (and artemisinin resistance in particular), and therefore prime candidates for further analyses. In one of the shared segments we found […]

Looking at figure Table S5, it seems the evidence that its an independent haplotype seems to come only from the right hand flank of the gene? Am I correct in this? If so is it mentioned and discussed in the text? Could it possibly be a recombinant?

The analysis shown in Fig. S5 aims to identify where we find the haplotypes that have the longest stretch of identity with respect to the C580Y mutants. We observe that, on the left-hand flank, haplotypes with the same alleles as the C580Y mutants are found in both Cambodia and Wewak; on the right-hand side, however, similar haplotypes are only found in Wewak.

Had the left-hand side haplotype only occurred in Cambodia, and not in Wewak, we could have reasonably proposed that this was an imported haplotype that had recombined with a local parasite. However, since there are several (kelch13 wild-type) Wewak parasites carrying the same haplotype, and some of these possess the longest stretches of identity when the two flanks are combined, the most parsimonious hypothesis is that the C580Y mutation has emerged on a locally-circulating haplotype.

The question remains as to why the C580Y mutants' left-hand flank allele sequence is so similar to those in Cambodia, and hence whether the haplotype is indeed "independent", as the reviewer asks. This is a question that we are not in a position to fully answer in this study, and on which we preferred to avoid speculation. However, for the benefit of the reviewer, we should point out that this left-flank sequence (a) in Cambodia is not only associated with C580Y, but also with other *kelch13* variants; and (b) is also seen in Wewak wild-type *kelch13* parasites. This suggests that, even if this flanking haplotype originates from Southeast Asia, its importation is likely to have occurred long before the C580Y mutation was acquired. Hence, this analysis provides no evidence of a recent importation of C580Y from Southeast Asia, which was the aim.

We hope this explanation is sufficient. This point is somewhat peripheral to the manuscript, and discussing it at length may cause a loss of clarity, but we are happy to take the editors' advice.

This manuscript suggests that resistance evolves by multiple steps but an analysis of patterns over time in multiple locations would be really necessary to get a firm handle on this. As far as possible, it would be nice if this discussion could be walled off as more speculative. Are there specific predictions about

the specific patterns associated with resistance loci that the authors would like to make that could be investigated in a global way?

> We have revised completely the paragraph in question. While we have attempted make it clear what the evidence supports, we felt it was important to retain the hypothesis of an ongoing evolutionary process, since it suggests novel approaches to genetic surveillance.
>
> > Taken together, the above results frame the Wewak C580Y mutants in a complex evolutionary context. This *kelch13* mutation has emerged independently in New Guinea, and was found not on an ordinary Wewak genetic background, but on one that has acquired a patchwork of sizeable genomic segments from another population. These acquisitions have introduced genomic regions that were likely to be under selection in Papua Indonesia, and contain alleles new to PNG, which have been associated to drug resistance in other parts of the world. This strongly suggests that these mutants are the product of a process driven by recombination events and selection under artemisinin drug pressure, and that the *kelch13* C580Y mutation is a component of a complex constellation of genetic changes, rather than a standalone mutation. […]
> >
> > There is evidence that an analogous evolutionary process has taken place in the GMS where *kelch13* mutations emerged in parasites that had acquired a complex set of genetic changes. Several of these changes were present before ACT treatment failures occurred, and it is possible that a similar process is ongoing in New Guinea. It is likely that these additional mutations play a compensatory role: although the C580Y mutation alone can confer an RSA phenotype shift, it is also likely to introduce a fitness cost deriving from reduced growth at ring stage, which could prevent mutant parasites from thriving in the field. Further studies will be needed to establish whether there is a common pattern to these mutations, which could be used to detect the emergence of resistant strains from genomic surveillance data before *kelch13* mutations become established and clinical failures start to occur.

Reviewer 2

Part I - Summary

Please use this section to discuss strengths/weaknesses of study, novelty/significance, general execution and scholarship.

Reviewer #2: This is important and exciting work from arguably the world leaders in evolutionary genomics and molecular epidemiology of malaria parasites and drug resistance. The work is impressive both in terms of the study/data/analysis and in terms of the thoughtful presentation. However, while what the authors DO describe is compelling, there are important things they are not describing and putting in broader context. They stop short of using their study to address important unresolved questions about Art-R. Stopping short of clarity would be a missed opportunity to make the most of this work to provide a template that can serve to guide the field in the important and challenging area of artemisinin drug resistance (Art-R) evolution, particularly as relates to surveillance and to have a coherent scheme for decisions and policymaking. To this end, what does this work indicate for researchers on the front lines who are monitoring for emerging Art-R? The authors are having it both ways.

> The reviewer is raising the bar of expectations from this work, which we take to be a very positive reaction. By saying that we “are having it both ways”, we believe the reviewer is implying that our observations have the potential to be the foundation of a framework for supporting policy decisions, and we are shying away from the opportunity. While we are thrilled

that such potential is perceived, reality is simpler: at this stage, it is not possible to transform insights into the evolution of a specific resistant strain into universally applicable criteria for the surveillance of drug resistance.

Currently criteria for triggering public health responses to emerging ART-R, defined by the World health Organization (WHO 2014), require clinical evidence of artemisinin resistance (ART-R), and consider ART-R to be confirmed only when a significant proportion of patient fail treatment. In vitro and genetic evidence (such as prevalence of kelch13 mutations) are considered as evidence only for suspecting ART-R. Since policy changes are massively demanding for national malaria control programmes, it is understandable that conservative criteria are used. However, reliance on clinical phenotypes leaves public health "on the back foot" because clinical failure is generally a signal of advanced levels of resistance- especially with combination therapies like ACT, which typically fail only when there is resistance to all component drugs.

Over the last few years, the emergence of resistance to artemisinin and its ACT partner drugs have given us the first opportunities ever to observe the evolutionary process in action. It is important to emphasize that we do not fully understand the details yet. We share the referee's belief that the identification of patterns will allow the development of tools that can detect early signs of drug resistance emergence, which may prevent the onset of failures altogether. The present manuscript shows evidence that there are *P. falciparum* strains in New Guinea that not only carry the most common marker of ART-R, but also exhibit signatures of an underlying evolutionary process that is not observed in other local parasites. However, we still have insufficient data to develop robust rules that can be applied in surveillance. In this context, the referee may be interested in ongoing efforts such as the GenRe-Mekong genetic surveillance project (https://www.medrxiv.org/content/10.1101/2020.07.23.20159624v1), which aim to continually sequencing parasite genomes and uncover patterns that reveal signals of selection which can inform public health response.

What is/are the signature that matters? Is K13 (kelch) all we need to know? Or is that the final step to Art-R and what matters is a source of 'background' with specific features?

That is an insightful question that touches the core question of which criteria to use to trigger public health responses. There is a mounting body of experimental evidence indicating that *kelch13* mutations are indeed causal markers of slow parasite clearance, the type of resistance that we observe in Southeast Asia. They are also predictors of treatment failure rate, especially when accompanied by markers of resistance to partner drugs. Therefore, they are effective surveillance tools for predicting clinical outcomes- hence their incorporation in the WHO criteria and various surveillance platforms.

In addition, there is also substantial evidence to indicate that, although *kelch13* mutations may be critical (and perhaps even sufficient) for slow parasite clearance, it does not act alone. Several other mutations have been implicated in GWAS, and there is evidence of expression changes in pathways which do not involve *kelch13*. This should not be surprising: if a single mutation were sufficient, drug resistant strains would emerge and establish themselves very rapidly, rather than take years. Birnbaum *et al.* (later quoted by the reviewer) showed that *kelch13* mutations restrict ring-stage endocytosis, essentially stunting parasite growth. It is very unlikely that such changes would produce a competitive population, unless other major

functional adjustments. It is easy to see that these changes may occur in pathways that are not obviously related to drug response.

Many questions emerge from the above. What changes are necessary for *kelch13* mutations to be sustained in a population? Will the same changes be needed in different parts of the world where the circulating genomes are substantially different? Is there a time sequence in which they are acquired? Thanks to what selective advantage? Right now, we have no certain answers to these questions. Still, here we report some unique features of these three Wewak parasites, which indicate that "business is not as usual": (a) they possess a C580Y mutation that has emerged independently in New Guinea; (b) their genome contains a patchwork of sizeable acquired genomic segments from a distant source population on top of a "local" genetic background; and (c) some of these segments have been under selection in the source population. The fact these segments contain newly acquired alleles that were associated with resistance elsewhere is strong circumstantial evidence that these genetic variants are candidates for the functional components that support the *kelch13* mutation. We believe this is sufficient evidence to suggest there is an ongoing evolutionary process, that the strain should be monitored, and that the highlighted genetic variants should be further investigated.

In the Discussion, we have re-written the paragraph that deals with the complex genetic background, in order to clarify some of these aspects:

> Taken together, the above results frame the Wewak C580Y mutants in a complex evolutionary context. This *kelch13* mutation has emerged independently in New Guinea, and was found not on an ordinary Wewak genetic background, but on one that has acquired a patchwork of sizeable genomic segments from another population. These acquisitions have introduced genomic regions that were likely to be under selection in Papua Indonesia, and contain alleles new to PNG, which have been associated to drug resistance in other parts of the world. This strongly suggests that these mutants are the product of a process driven by recombination events and selection under artemisinin drug pressure, and that the *kelch13* C580Y mutation is a component of a complex constellation of genetic changes, rather than a standalone mutation. […]
>
> There is evidence that an analogous evolutionary process has taken place in the GMS where *kelch13* mutations emerged in parasites that had acquired a complex set of genetic changes. Several of these changes were present before ACT treatment failures occurred, and it is possible that a similar process is ongoing in New Guinea. It is likely that these additional mutations play a compensatory role: although the C580Y mutation alone can confer an RSA phenotype shift, it is also likely to introduce a fitness cost deriving from reduced growth at ring stage, which could prevent mutant parasites from thriving in the field. Further studies will be needed to establish whether there is a common pattern to these mutations, which could be used to detect the emergence of resistant strains from genomic surveillance data before *kelch13* mutations become established and clinical failures start to occur.

And what about the phenotype limitations of this study? Without the single C580Y parasite for which they have RSA data (indeed, it is their most resistant RSA of all successfully measured), the message of this paper would be largely muted. And they give nearly no consideration to the 5 RSA resistant parasites that carry a kelch wild type (WT). Is the phenotype uninteresting in this case? Are these 2/3 of the way to resistant (but still lacking the kelch mutation).

Clearly, the RSA results lend more weight to the claim that the parasites with the C580Y mutation are ART-R but, in epidemiological terms, the identification of acquired genomic regions holds the same interest regardless.

Concerning the remaining parasites with RSA>1, we would have liked to attempted to analyse them in more detail, but we only had genomic data for one of them (RSA=1.613, the 5th highest). This is unfortunate but not necessarily surprising, since several samples yielded insufficient DNA for sequencing. This parasite clustered with several other Wewak samples, and in the PCoA plot (Fig. 2A) it falls within the group of wild-type PNG samples. In the Admixture analysis (Fig. 2B) it is predicted as being of fully PNG ancestry (all blue). It shares high levels of IBD with four other Wewak parasites; we have RSA results for two of these (RSA=0 for both). There was no headline result, so we did not discuss this in the manuscript.

Part III – Minor Issues: Editorial and Data Presentation Modifications
Please use this section for editorial suggestions as well as relatively minor modifications of existing data that would enhance clarity.

Reviewer #2: Broadly, there are unaddressed items that will leave an uninitiated reader confused. For example:

The growing body of literature implies that a mutation in K13, specifically the most successful C580Y mutation, is the Art-R determining mutation. Recent work by Birnbaum et al reinforced this with their compelling (and surprising) functional basis for Art-R. The emerging story would thus indicate that Art-R evolution is simple, sweep signatures should be generally hard, clinical phenotypes unambiguous and that K13 mutations is the only relevant surveillance. This manuscript nicely illustrates that it is more nuanced, but stops short of taking on any of these points directly. Because widespread Art-R would be a devastating blow to malaria control, researchers around the world are groping for how best to identify emerging resistance and rapidly respond with smart adjustments to local policies.

Our earlier comments addressed these points. As the reviewer says, the drug resistance story is bound to be more nuanced than the emergence of a single mutations. However, monitoring kelch13 variants (and C580Y in particular) is undoubtedly valuable and informative for a range of public health applications. We hope our results will help lay the foundations for new surveillance methods that will identify the underlying evolutionary signatures of drug resistance emergence.

Why is there no treatment failure or evidence of delayed clearance on the island? Is this a feature of the local malaria? The specific activities in the clinics? The phenotypes per se? e.g. Are these ex vivo RSA data reliable? Is RSA a reliable surrogate for clearance times? And are both underpinning drug failure? Are there unique aspects to malaria, clinical access, reporting that could influence this interpretation (e.g. of hidden emerging resistance)?

In general, clinical failures would not solely depend on artemisinin resistance, since artemisinin is used in combination with a partner drug that is intended to complete the parasite killing action. Thus, it is likely that treatment failures will become numerous only when resistance to the ACT partner drug emerges alongside resistance to artemisinin. In the GMS, failure rates rapidly escalated when piperaquine resistance combined with artemisinin resistance, years after slower parasite clearance rates were first observed. At this stage, clinical failures may not occur in New Guinea, but experience in the GMS suggests they could happen in the near future. These points are made in the third paragraph of the Discussion.

At present, RSA is the *in vitro* test that best correlates with elevated parasite clearance half-life [Witkowski et al. (2013) Lancet Infect Dis.13(12):1043-9.]. It was specifically developed to test artemisinin response, since the IC50 tests used for other drugs are poor predictors of slow parasite clearance. RSA focus on ring-stage development is consistent with experimental results (including the Birnbaum *et al.* paper) showing that survival from artemisinin is largely dependent on a ring stage-specific phenotype.

The gold standard for characterizing the slow parasite clearance phenotype is to measure the artemisinin clearance half-life from 6-hourly parasite counts during treatment. This is a very resource-intensive and expensive testing method that is only performed in certain research studies (e.g. the TRAC study, see Ashley EA et al. (2014) NEJM); we are not aware of any that have been conducted in New Guinea.

Are the findings of Birnbaum et al relevant to understanding/interpreting these data? Not relevant at all? Why?

Studies like Birnbaum's are extremely valuable, because they provide clarity on the role of *kelch13* mutations, but also because they show that the pathway involved is hugely impactful for parasite development, giving us a key for interpreting associations between ART-R and other, seemingly unrelated, pathways. From their results, one should reasonably expect *kelch13* mutations to be underpinned by several genetic changes affecting multiple pathways. We are now explicitly stating this in the Discussion.

One very important finding is that *kelch13* appears to be the only protein in the endocytosis pathway that causes ring-stage specific phenotype changes, which could explain why ART-R was not associated with changes in other genes involved in that pathway. Hence, we should not be surprised that those genes do not feature in the constellation of mutations that accompany C580Y- as is the case in our results.

Timiki is crucial for reconstructing the current epidemiological situation in Wewak C580Y mutants. The authors give very little information about what is special about these parasites, e.g. the drug (combination) of choice in that region, duration (and sequence), intensity of selection, clinical situation, the possible source of introduction of this background.

In the recent past, Timika has harboured high levels of multidrug resistant *P. falciparum* and *P. vivax* (Ratcliff A, *et al. Trans R Soc Trop Med Hyg* 2007; **101**: 351-9; Sumawinata IW*, et al*. *Am J Trop Med Hyg* 2003. **68**:416-20). Surveys and clinical drug efficacy studies conducted between 2004 and 2006 demonstrated 48% treatment failure following CQ plus sulfadoxine-pyrimethamine (SP) for *P. falciparum and* 65% failure against CQ monotherapy for *P. vivax* malaria (Ratcliff A, et al. *Trans R Soc Trop Med Hyg* 2007; **101**(4): 351-9). In March 2006, local antimalarial guidelines were revised to recommend that all patients with uncomplicated malaria (due to any *Plasmodium* species) should be treated with dihydroartemisinin-piperaquine (DHP). The Timika population has therefore experienced high levels of DHP pressure for over 14 years. A recent open-label drug efficacy study for uncomplicated malaria (conducted between March 2015 and April 2016) demonstrated that the efficacy of DHP was 98% in patients presenting with *P. falciparum* malaria, with no evidence of K13 mutations associated with artemisinin resistance or increased copy number of *plasmepsin*2-3 (Poespoprodjo JR, et al. *Am J Trop Med Hyg* 2018; **98**(3): 677-82). A microsatellite-based study, evaluating parasite genetic diversity and structure

in Timika over the same period, identified a distinct parasite subpopulation that emerged in the late post-ACT period (2016-2017), rapidly rising in frequency to 100% of infections, a pattern typical of a population under selection (Pava Z, et al. *PLoS Negl Trop Dis* 2020; **14**(5): e0008295).

We have modified the discussion to introduce a couple of salient points:

> […] However, a substantial proportion of C580Y mutants' genome consists of long haplotypes shared with many of the Timika parasites, suggesting these segments were acquired from a common source population, strongly connected to the Papua Indonesia parasites. Furthermore, the length and frequency of the shared haplotypes suggests that they contain regions under selection in that source population. This may be consistent with a recent report that a distinct parasite subpopulation has emerged in Timika and rapidly risen in frequency to 100% of infections in 2016-2017, potentially indicating a selective sweep, even though the frontline ACT is still reported to be efficacious.

The authors describe these genome segments as "recently acquired and possibly under selection"; explain this further (particularly the inference and role of selection, before and after introgression). I appreciate the authors hesitate to speak beyond their data, but there is room for more clarity about why this matters.

Please see an earlier response that deals with this point (first response, to Reviewer 1). We trust the changes provide the clarity requested by the reviewer.

Besides C580Y, it seems there are no other K13 mutations to mention?

The C580Y mutations in the three Wewak parasites were the only nonsynonymous changes in the relevant domains of kelch13 that we observed in New Guinea (including both PNG and Indonesia). This is stated in the Results.

It would be useful to more directly compare the findings here with a similar recent report from Guyana; what do these works say about expectations with respect to Art-R appearing in Africa? What information will be needed in future studies to get definitive actionable results. What is the recommendation based on this study for ongoing surveillance, in PNG and around the world?

We appreciate the referee's enthusiasm for finding common patterns that can be used to detect resistance emergence. We feel this is very important but, as stated in a previous reply, we are not at a stage where we can formulate rules. If common patterns underpin the emergence of resistance in Guyana, the GMS, New Guinea (and recently Rwanda), it would be beneficial to for these analyses to be joined up. However, the data available from different studies are not homogeneous- for example, whole genome data is not available in all cases, which renders these analyses difficult. Nevertheless, we think it is worth pursuing.

Other intriguing statements need clarification: "gradually acquired a complex ensemble of variants" (in what order? Can C580Y exist absent the background? Is the RSA phenotype shift caused by only C580Y?). Is this what the authors mean by "not a chance event"? i.e. that there is no C580Y by itself? But otherwise, it seems there is no specific mutation diagnostic of Art-R, but perhaps there is a signature emerging of some sort?

As the referee points out, it is quite hard to get to grips with the relationship between kelch13 and other accompanying genetic changes. Hopefully some of our previous answers have provided more clarity.

It has been shown that C580Y can be edited on a wild-type parasite, producing an RSA phenotype shift [Straimer et al. (2015) Science 347, 428–431]. Therefore, strictly speaking, C580Y can exist by itself and produces ART-R. However, that does not mean that such parasites could thrive in the field, as discussed in an earlier point. In the GMS, a genome-wide association study (GWAS) revealed a number of additional point mutations with highly significant association to clearance half-life [Miotto et al. (2015) Nat Genet 47(3):226-34]. These were found in genetically different parasites carrying different kelch13 mutations, and ART-R alleles were generally not observed in parasites lacking these additional mutations, leading to the conclusion that they predisposed parasites to the establishment of ART-R alleles ("not a chance event"). We have improved the discussion of these additional mutations in the Discussion section, e.g.

> It is likely that these additional mutations play a compensatory role: although the C580Y mutation alone can confer an RSA phenotype shift, it is also likely to introduce a fitness cost deriving from reduced growth at ring stage, which could prevent mutant parasites from thriving in the field. Further studies will be needed to establish whether there is a common pattern to these mutations, which could be used to detect the emergence of resistant strains from genomic surveillance data before *kelch13* mutations become established and clinical failures start to occur.

How can this method (e.g. IBD, key background components, K13 haplotypes) be systematically considered going forward, across different geographies by different groups? If groups are content to sequence kelch, will that provide the necessary real time information to adjust to newly arriving/emerging Art-R. The authors say (Art-R) "went undetected on the island". What is predicted? What is advised? The last sentence in the abstract implies this information will be useful to "to contain the spread"; explain a bit more how this would work.

As stated in previous answers, we believe we have strong evidence for an ongoing evolutionary process, which may be mirroring the process that occurred in the GMS. However, at present, we are not in a position to distil patterns that can be translated into surveillance tools for public health; this will require further work. We have put words to that effect in the Discussion.

These sentences from the Discussion are a good attempt, but are cryptic, with several opportunities to speak more specifically (comments interspersed).

"We propose that one or more New Guinean lineages have accumulated a complex genetic background through recombination events and selection under artemisinin drug pressure"- necessarily art pressure? Do particular partner drugs play a crucial role? All are CQR? Is that (or other selection histories) relevant?

In this context, we were referring to the fact that, where we identified a mutation associated to drug resistance, such as *ferredoxin*, *atg18* and *nif4*, they were association with response to artemisinin compounds. Although it is certainly likely that partner drugs are also causing selective pressures, we could show no specific evidence for that here.

“and that the kelch13 C580Y mutation is a component of a complex constellation of genetic changes, rather than a standalone mutation generated by a chance event“- what sort of component? essential, necessary? Must come last? due to fitness limitations? Is there a pre- or low-level resistance? “it appears that several of these changes were present before ACT treatment failures occurred”- my understanding is there is no report of treatment failure in PNG, is this referring to GMS? Does this scenario align with recent Guyana findings?

We acknowledge that some sentences may have been stated in a way that was less than clear. The section has been re-written, in a way that we hope answers these points.

“suggesting they provided improved fitness of the parasite population without major effects on clinical outcomes”- are you confident that clinics were well-tuned to identify this?

We understand from the local public health authorities and researchers operating in PNG (represented in the authorship) that clinical failures can be reliably identified; furthermore, new projects monitoring the efficacy of the frontline drugs are being carried out across the country.

“In scenarios where drug resistance requires a gradual build-up of genetic changes, clinical efficacy monitoring is only likely to detect resistance at an advanced stage of the evolutionary process when failures begin to occur”- do we have examples of this with other drugs/pathogens? major implications here about phenotypes…be clear what is needed

The problem of gradual genetic evolution of drug resistance, and the attainment of higher levels of resistance, is recognized in the context of many diseases. The introduction of the following article: Day T, Gandon S (2012) *The evolutionary epidemiology of multilocus drug resistance*. Evolution 2012; 66(5):1582-97 cites several examples.

Just for clarity, the problem highlighted here is not one of phenotypes, but rather one of detection methods. Clinical failure detection is the most widely accepted standard, but it is not sensitive to low levels of resistance (see Laufer MK, Curr Infect Dis Rep. 2009 11(1): 59–65).

“In the future, it is possible that novel analyses of evolutionary patterns, using genomic surveillance data, could allow earlier detection of drug resistance, and interventions ahead of clinical failure”- yes, this is the one that could move the field forward if the authors will speak more clearly…what would this have to look like?

See our previous replies to related questions.

Specific comments
Some additional details needed:
1. In figure 2B the fastStructure analysis shows a small subset of parasites from Wewak have as much or more Timiki ancestry as the C580Y parasites. Do any of the parasite in this subset also have the predominantly Timiki alleles at the same alleles that are identified in large sections from the C580Y parasites that are IBD with Timiki parasites? For example, in Figure 4C (the IBD plot for the region surrounding atg18) it appears that for some Wewak parasites, in addition to the C580Y parasite, that cluster with the Timiki parasites; however, this does not seem to be the case for flanking the pnp gene.

In fastSTRUCTURE, the C580Y mutants exhibit 33-35% from the Timika ancestral population. Two samples score a higher Timika ancestry proportion (40% and 42%), and a third is close (29%); other parasites have proportions <12%. All three samples were collected in 2017, like the C580Y mutants. In the PCoA these parasites are positioned close to the C580Y mutants- not surprising, because PC1 strongly correlates with genetic differences between Timika and PNG. Two of these samples (red circles) are visible in the PCoA plot to the right (42%) and left (29%) of the Wewak parasites (yellow circles). The third sample (40% proportion) is not visible because it is covered by the C580Y mutants, since it maps almost exactly to the same coordinates.

The question is whether the high ancestry proportion is caused by the same IBD segments as the C580Y mutants, or by a different allele pattern. We mapped out across the genome the proportion of Timika parasites that are in IBD with each of these three samples, and aligned the plots against the IBD proportions for the C580Y mutants (see plot below). It is clear that the two parasites with the highest proportion of Timika ancestry (blue and magenta plots) possess a genomic IBD pattern consistent with that of the C580Y mutants (red plot). Crucially, there is excellent correspondence between the IBD peaks in these parasites and those in the C580Y mutants. Although the third sample (green) is less similar, we can still observe IBD peaks corresponding to the C580Y mutants' loci on chromosomes 5,6,7,8 and 10.

Overall, we can hypothesize that at least two of these parasites are the product of the same process that produced the genetic background on which the C580Y mutation has emerged- i.e. they can be classified as being part of the same sub-population. Clearly, it is very interesting that members of this population are present both with and without the C580Y mutation. It may indicate that, at the time of collection, the process of C580Y acquisition was still ongoing in a population that was expanding into Wewak even without the benefit of the *kelch13* mutation.

This analysis enriches the story. We have added the figure to the Supplementary Material, and the following to the Results:

> We applied IBD analysis to other Wewak parasites, also collected in 2017, which lacked *kelch13* mutations but had high proportions of Indonesian ancestry (as determined by fastSTRUCTURE). We found that the two samples with the highest Indonesian ancestry proportions (42% and 40%) show a near-identical pattern of high-IBD regions to that of the C580Y mutants; a third parasite with a lower degree of Indonesian ancestry (29%) also harboured several corresponding high-IBD regions (Figure S6). It therefore appears that parasites carrying very similar sets of acquired genomic regions, both with and without the C580Y mutation, circulated simultaneously in Wewak.

And the following to the Discussion:

> This strongly suggests that these mutants are the product of a process driven by recombination events and selection under artemisinin drug pressure, and that the *kelch13* C580Y mutation is a component of a complex constellation of genetic changes, rather than a standalone mutation. The presence of parasites carrying a similar compendium of acquired genomic segments, but no *kelch13* C580Y mutation, supports the hypothesis that the latter is a very recent acquisition on a circulating genetic background.

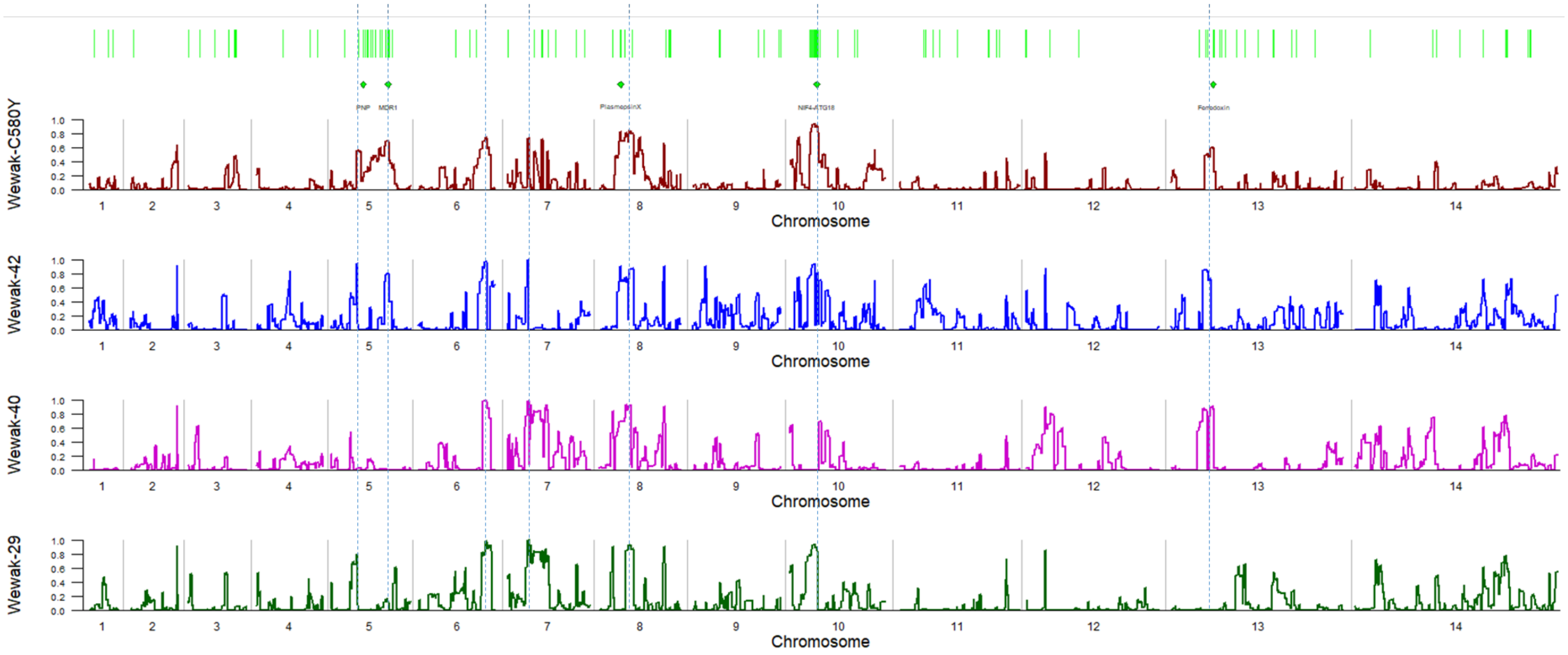

Figure S6 – Correspondence between IBD regions in Wewak C580Y mutant and those in Wewak kelch13 wild-type parasites with high proportion of Timika ancestry. The plots show, across the 14 nuclear chromosomes, the proportion of IBD pairs between Timika and the Wewak C580Y mutants (red, top), and three Wewak samples that showed a high proportion of common ancestry with Timika: one with 42% Timika ancestry (blue), one with 40% (magenta) and one with 29% (green). The top panel shows vertical green bars marking highly differentiated positions where the C580Y mutant carry a Timika-like allele. Green diamond markers show the location of some notable drug resistance-related alleles identified in our analysis. Vertical dotted lines act as visual guides to show correspondences.

2. Do the authors think these variants have been under selection in Timiki? They give little information about 'why Timiki' as the source of background, e.g. in terms of drug selection history, current ACT, any clinical information.

> We report strong circumstantial evidence for such selection. However, we are aware of ongoing genetic analyses in Indonesia that will provide greater clarity. In a previous reply, we have provided details of Timika's history of drug resistance.

3. Define clinical outcomes in both PNG and Indonesia Papua…has there been treatment failure? Clearance rates were not measured, true? So the authors can't speak to the impact of these mutations on clearance rate specifically.

> The most recent clinical results do not report significant levels of treatment failures- neither in Timika nor in Wewak.
>
> It is true that clearance rates were not measured on any of these parasites; however, the RSA test has been shown to give results that are correlated to clearance half-life [Witkowski et al. (2013) Lancet Infect Dis.13(12):1043-9.]. Hence we predict that the Wewak C580Y parasites would exhibit slow parasite clearance.

4. It needs to be addressed more directly (as it is highly relevant to surveillance)… if it is true that there is no treatment failure, including for the kelch mutants, there is not a strong ground based on these studies alone to focus entirely on the (assume) the role of kelch, and e.g. to discount the other RSA resistant kelch WT parasites, yet there is no mention of these parasites (or specific consideration of their Timiki IBD; are these one step toward resistant? Awaiting only a C580Y mutation?

> This was covered in earlier responses. Under an ACT regime, treatment failures become common when resistance to both drugs is present. This does not appear to be the situation here. However, that does not mean the parasites are not resistant to artemisinin. Although the infections are ultimately cleared, artemisinin resistant parasites survive longer in greater number and have a greater chance of transmitting and of building up resistance to the partner drug.
>
> The remaining high-RSA parasites were not "discounted": unfortunately, we had insufficient data (only one individual) to analyze them further. We hope subsequent sample collections may inform us further.

5. In Table S3 they have survival rate greater than 0% but the RSA cutoff is 1% and they state that in line 121, why are these not consistent? Do the RSA resistant kelch WT share any specific mutations they list in figure S6 between the C580Ys? There might be value to highlighting these in the PCoA in Fig2. Are there candidate parasites that 'bridge the gap' between the clusters from the two populations? Do the samples H5, H13, H46 and H48 parasites (lines 172-174) share sequence similarities?

> The RSA>1% cutoff was established as correlating with a clearance halflife threshold of ~5hrs [Witkowski et al. (2013) Lancet Infect Dis.13(12):1043-9.], and is commonly used to classify ART-R parasites. It is not a perfect classifier, but the correlation is highly significant.

We only have genomic data for one of the parasites with RSA>1%, and therefore it was not possible to identify candidate mutations, since each individual typically carries hundreds of alleles that differentiate it from the rest of the local population. Looking for the mutations listed in Table S6 would only be meaningful if we thought those mutations affect the RSA phenotype- but we have no reason to suspect this is the case, since we expect them to be mostly compensatory. This is also the only individual with RSA>1% we could include in the PCoA plot, and it falls in the midst of the Wewak population, not amongst the individuals that are mid-way between the two major populations.

The samples with the H5, H13, H46 and H48 are not especially similar to each other. We have genomic data for five of them, including at least one per haplotype. In the PCoA plot, they cluster with the bulk of PNG samples, nor particularly close to each other, with the exception of one of the H13 samples, which is the parasite with 40% Indonesian ancestry mentioned earlier. We do not feel there is anything worth reporting about this.

Minor comments:

Lines 165-176, 177-183, 201-206 show that the haplotype didn't come from Cambodia; does this rule out other SEA sources?

Clearly, we cannot rule out every possible origin in the GMS- the Cambodia population was chosen as it is the most representative of the most common C580Y haplotypes in that region. Given that the longest identical flanking haplotypes circulate in wild-type parasites in New Guinea, recent importation from GMS populations that we have not analysed is a less likely explanation than local emergence.

Line 215, might be better to say "chosen" rather than "selected".
For TableS6 Mutation Frequency, ID needs to be Timika.
Line 278 – showed that one Wewak mutant, not "mutants", are resistant to art.

These have been corrected.

Line 201-204/figure 4. They have 3 Wewak C580Ymutants but only show 2 points in their clusters. Enlarge the circles and squares to make clear what is grayed out for the figure key to be more clear.

Indeed, only two C580Y samples could be included in that analysis, the remaining one did not meet the missingness criteria. We now included some explanatory text in the Fig. 5 legend: "note that only two mutants were included in this analysis, due to high degree of genotype missingness in the third mutant."

For Fig 5 and lines 207-227 indicate Timika segments are selected by fitness advantage or drug pressure. How do the authors envision their Fig 5 will look in the future? Will those section of chromosome that show high IBD with Timika parasite be whittled down even further, if those mutations work better with C580Y to make drug resistant parasites? How would that graph look with those WT parasites?

Great question. Assuming the process continues under artemisinin pressure, one scenario is that the regions in IBD with the C580Y parasites will gradually narrow around the selected mutations,

as a result of continued recombination. The selected mutations will remain at a high frequency, while other "hitchhiking" mutations may gradually become less common. This should only be considered one of several possible scenarios: it is entirely possible the course of the process will be changed by other factors, for example emergence of resistance to a partner drug.

Can efficacy studies detecting delayed PC1/2 demonstrate advanced resistance? A point they could make that would help this approach is whether there is enough new cases happening in PNG to make efficacy studies happen. If there isn't, the only way to really monitor resistance is to do in vitro RSA and sequencing?

As mentioned in another reply, measuring clearance half-life is a very onerous task, which is not normally conducted by therapeutic efficacy studies, which tend to focus on parasite levels on Day 3. Genetic surveillance and in vitro testing of suspected resistance can be used to justify in-depth investigation of the in vivo phenotypes. This is not current policy yet, although malaria control programmes are continually gaining confidence in the potential of such approaches.

These low transmission areas seem susceptible to Art-R generation. It would be useful to consider whether this information will be relevant for Art-R in Africa.

Indeed. A recent report from Rwanda (Uwimana, A., et al. (2020) *Emergence and clonal expansion of in vitro artemisinin-resistant Plasmodium falciparum kelch13 R561H mutant parasites in Rwanda*. Nat Med. https://doi.org/10.1038/s41591-020-1005-2) shows that this may already be happening. We are doing our best to share information note that Dr P Ringwald of WHO is an author both on the present manuscript and the Rwanda paper.
